# Marital status and survival of patients with kidney cancer

**DOI:** 10.18632/oncotarget.21029

**Published:** 2017-09-16

**Authors:** Tingru Miao, Yufeng Li, Xiaoli Sheng, Dingguo Yao

**Affiliations:** ^1^ First Affiliated Hospital of Zhejiang Chinese Medical University, Hangzhou, China

**Keywords:** kidney cancer, marital status, SEER database, survival analysis

## Abstract

**Background:**

The relationship between marital status and prognosis of kidney cancer has not been explored in detail. In this study, we aimed to investigate the impact of marital status on survival outcomes in kidney cancer.

**Methods:**

We used the Surveillance, Epidemiology and End Results (SEER) program to identify 112860 patients with kidney cancer diagnosed in 2004 through 2013. Kaplan-Meier methods and multivariable Cox regression models were used to analyze the influence of marital status on overall survival (OS) and cancer-specific survival (CSS).

**Results:**

Married patients had better 5-year OS and CSS compared with patients who were divorced/separated, widowed, and single. After adjusting for known confounders, unmarried patients were at greater risk of overall and cancer-specific mortality, especially the widowed. Moreover, subgroup analysis showed that married still had better prognosis across different SEER stages, ages and sexes.

**Conclusions:**

Our study revealed that marriage is associated with better outcomes of both OS and CSS in kidney cancer patients.

## INTRODUCTION

Marital status has been considered as an independent prognostic factor of survival in several types of cancer [1–4]. It is well known that social factors may influence the outcome of various diseases [5]. Marital status is regarded as an important social support factor and can greatly affect the patient’s emotions, life style, and financial status [6, 7]. Previous studies have shown that being married has a protective role in health and cancer survival compared with being unmarried [8, 9].

Kidney cancer accounts for nearly 2% of all cancers, and there were an estimated 338,000 newly diagnosed cases and 143,000 deaths due to kidney cancer in 2012 [10]. In addition, 70% of new cases occurred in developed countries. Incidence and mortality rates of kidney cancer have been increasing in most countries over the past 30 years [11]. It is well-established that cigarette smoking, obesity and hypertension are risk factors for renal cell cancer [12]. However, little is known regarding the association between marital status and survival of patients with kidney cancer. In this study, we used data from the Surveillance, Epidemiology, and End Results (SEER) program to explore the impact of marital status on survival outcomes of kidney cancer patients.

## RESULTS

### Patient characteristics

We investigated a total of 112860 kidney cancer patients in the SEER database from 2004 to 2013, including 71549 (63.4%) male and 41311 (36.6%) female. Most patients are white (82.3%). According to marital status, 71328 (63.2%) were married at diagnosis, and 41352 (36.8%) were unmarried, including 11896 (10.5%) divorced/separated, 13160 (11.7%) widowed, and 16467 (14.6%) single. Compared with unmarried patients, the married patients were more likely to be diagnosed at an earlier stage and receive treatment. The widowed group had the highest percentage of women, the most elderly patients, and the lowest proportion of stage I and localized patients compared with the other groups and more were less likely to receive treatment. The baseline clinicopathological features are shown in Table 1.

**Table 1:** Baseline clinicopathological features of kidney cancer patients in SEER database

Characteristic	Total	Married	Divorced/Separated	Widowed	Single	P value
	112860(100)	71328(63.2)	11896(10.5)	13160(11.7)	16476(14.6)	
**Sex**						<0.001
Male	71549(63.4)	50344(70.6)	6697(56.3)	3973(30.2)	10535(63.9)	
Female	41311(36.6)	20984(29.4)	5199(43.7)	9187(69.8)	5941(36.1)	
**Age**						<0.001
<60	43627(38.7)	27709(38.8)	5307(44.6)	847(6.4)	9764(59.3)	
60-70	31801(28.2)	21745(30.5)	3859(32.4)	2347(17.8)	3850(23.4)	
70-80	24332(21.6)	15603(21.9)	2093(17.6)	4607(35.0)	2029(12.3)	
>80	13100(11.6)	6271(8.8)	637(5.4)	5359(40.7)	833(5.1)	
**Race**						<0.001
White	92909(82.3)	60549(84.9)	9474(79.6)	10975(83.4)	11911(72.3)	
Black	13076(11.6)	6045(8.5)	1920(16.1)	1535(11.7)	3576(21.7)	
American Indian/ Alaska Native	952(0.8)	470(0.7)	125(1.1)	117(0.9)	240(1.5)	
Asian/Pacific Islander	5324(4.7)	3853(5.4)	318(2.7)	505(3.8)	648(3.9)	
Unknown	599(0.5)	411(0.6)	59(0.5)	28(0.2)	101(0.6)	
**Grade**						<0.001
High/Moderate	51438(45.6)	33725(47.3)	5421(45.6)	4745(36.1)	7547(45.8)	
Poor/Undifferentiation	29071(25.7)	19216(26.9)	3052(25.7)	2604(19.8)	4199(25.5)	
Unknown	32351(28.7)	18387(25.8)	3423(28.8)	5811(44.2)	4730(28.7)	
**TNM**						<0.001
I	63996(56.7)	41329(57.9)	6733(56.6)	6552(49.8)	9382(56.9)	
II	9309(8.2)	5943(8.3)	986(8.3)	870(6.6)	1510(9.2)	
III	14165(12.6)	9441(13.2)	1358(11.4)	1511(11.5)	1855(11.3)	
IV	17801(15.8)	10524(14.8)	2044(17.2)	2552(19.4)	2681(16.3)	
Unknown	7589(6.7)	4091(5.7)	775(6.5)	1675(12.7)	1048(6.4)	
**SEER Stage**						<0.001
Localized	76192(67.5)	48989(68.7)	8025(67.5)	7877(59.9)	11301(68.6)	
Regioned	16162(14.3)	10653(14.9)	1555(13.1)	1810(13.8)	2144(13.0)	
Distant	16786(14.9)	9912(13.9)	1932(16.2)	2394(18.2)	2548(15.5)	
Unstage	3720(3.3)	1774(2.5)	384(3.2)	1079(8.2)	483(2.9)	
**Therapy**						<0.001
Surgery, radiation or both	94075(83.4)	62033(87.0)	9803(82.4)	8581(65.2)	13658(82.9)	
No surgery, radiation	17823(15.8)	8845(12.4)	1980(16.6)	4354(33.1)	2644(16.0)	
Unknown	962(0.9)	450(0.6)	113(0.9)	225(1.7)	174(1.1)	

### Impact of marital status on overall survival in the SEER database

**Figure 1:**
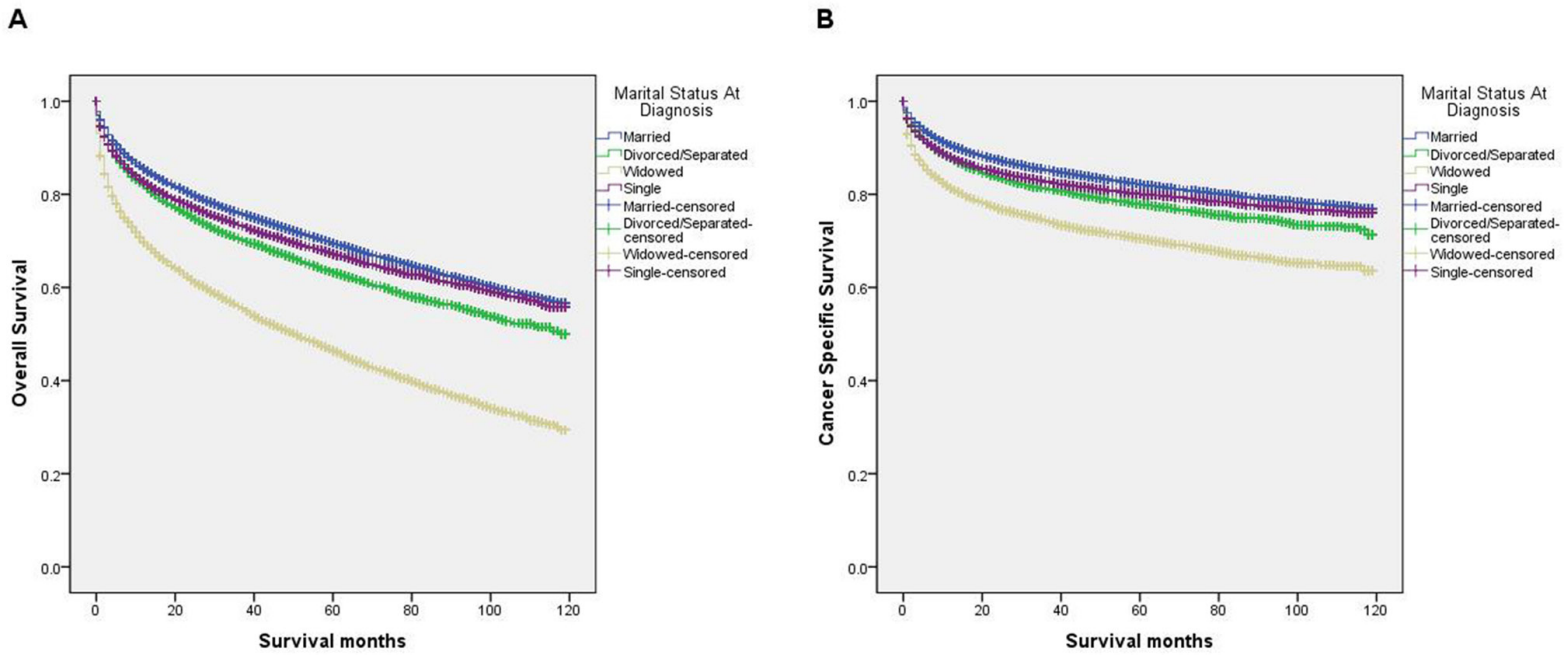
Survival curves in kidney cancer patients according to marital status **(A)** Overall survival: χ2 = 3122.46, P < 0.001. **(B)** Cancer specific survival:χ2 =1039.78, P < 0.001.

We performed a Kaplan-Meier analysis to reveal the difference in overall survival (OS) according to marital status (log-rank test p<0.001) (Figure 1A). The 5-year OS rate was 69.5% in the married group, 63.2% in the divorced/separated group, 46.4% in the widowed group, and 67.2% in the single group. In addition to marital status, the results of the Kaplan-Meier analysis indicated that sex, age, race, grade, TNM stage, SEER stage, and therapy were significantly associated with OS in these patients. Cox regression was also used to adjust the variables mentioned above in the multivariate analysis, and marital status was found to be an independent prognostic factor for overall survival (married, reference; divorced/separated, HR=1.25, 95% CI 1.21-1.30, P<0.001; widowed, HR=1.25, 95% CI 1.21-1.29, P<0.001; single, HR=1.18, 95% CI 1.14-1.22, P<0.001) (Table 2).

**Table 2:** Univariate and multivariate analysis for overall survival (OS) in kidney cancer patients

Variables	5-year OS	Univariate analysis		Multivariate analysis		
		Log rank Χ^2^	P value	HR	95% CI	P value
**Sex**						
Male	64.70%	48.23	<0.001	Reference		
Female	67.50%			0.89	0.87-0.91	<0.001
**Age**						
<60	77.90%	12093.60	<0.001	Reference		
60-70	68.70%			1.38	1.34-1.42	<0.001
70-80	58.60%			1.84	1.78-1.89	<0.001
>80	32.20%			2.61	2.52-2.71	<0.001
**Race**						
White	65.60%	136.86	<0.001	Reference		
Black	65.00%			1.07	1.04-1.11	<0.001
American Indian/Alaska Native	63.40%			0.97	0.87-1.09	0.587
Asian/Pacific Islander	68.50%			0.87	0.82-0.91	<0.001
Unknown	90.50%			0.31	0.23-0.42	<0.001
**Grade**						
High/Moderate	83.10%	16886.37	<0.001	Reference		
Poor/Undifferentiation	60.50%			1.70	1.65-1.75	<0.001
Unknown	42.00%			1.65	1.60-1.71	<0.001
**TNM**						
I	83.10%	59552.13	<0.001	Reference		
II	76.80%			1.43	1.36-1.50	<0.001
III	63.10%			0.83	0.75-0.93	0.001
IV	10.30%			2.04	1.81-2.30	<0.001
Unknown	42.90%			1.54	1.44-1.65	<0.001
**SEER Stage**						
Localized	81.60%	61354.67	<0.001	Reference		
Regioned	58.90%			2.73	2.46-3.03	<0.001
Distant	9.40%			4.27	3.79-4.81	<0.001
Unstage	25.90%			1.69	1.57-1.83	<0.001
**Marital Status**						
Married	69.50%	3122.46	<0.001	Reference		
Divorced/Separated	63.20%			1.25	1.21-1.30	<0.001
Widowed	46.40%			1.25	1.21-1.29	<0.001
Single	67.20%			1.18	1.14-1.22	<0.001
**Therapy**						
Surgery, radiation or both	75.00%	39178.54	<0.001	Reference		
No surgery, radiation	17.60%			2.49	2.41-2.56	<0.001
Unknown	30.40%			2.30	2.11-2.50	<0.001

### Impact of marital status on cancer-specific survival in the SEER database

Similarly, the survival curve showed that cancer-specific survival (CSS) among groups with different marital status was significant (log-rank test P<0.001) (Figure 1B). The 5-year CSS rates for the married group, the divorced/separated group, the widowed group and the single group were 82.1%, 77.8%, 70.4% and 80.1%, respectively. Among clinicopathological variables, sex, age, race, grade, TNM stage, SEER stage, therapy, and marital status were identified as risk factors for predicting CSS based on a Kaplan-Meier analysis. When a multivariate analysis with Cox regression was performed, marital status was confirmed to be an independent prognostic factor for kidney cancer prognosis (married, reference; divorced/separated, HR=1.20, 95% CI 1.15-1.26, P<0.001; widowed, HR=1.24, 95% CI 1.19-1.30, P<0.001; single, HR=1.14, 95% CI 1.09-1.19, P<0.001) (Table 3). In addition, age (≥ 60y), poor/undifferentiated, TNM stage, SEER stage, and no surgery and/or radiotherapy were associated with poorer CSS.

**Table 3:** Univariate and multivariate analysis for cancer specific survival (CSS) in kidney cancer patients

Variables	5-year CSS	Univariate analysis		Multivariate analysis		
		Log rank Χ^2^	P value	HR	95% CI	P value
**Sex**						
Male	79.50%	13.26	<0.001	Reference		
Female	81.00%			1.01	0.98-1.04	0.524
**Age**						
<60	84.10%	2813.62	<0.001	Reference		
60-70	81.20%			1.07	1.03-1.11	0.001
70-80	79.00%			1.14	1.09-1.19	<0.001
>80	63.30%			1.52	1.45-1.60	<0.001
**Race**						
White	79.90%	79.39	<0.001	Reference		
Black	81.60%			0.99	0.95-1.04	0.736
American Indian/Alaska Native	75.90%			1.00	0.87-1.16	0.970
Asian/Pacific Islander	78.90%			0.98	0.92-1.05	0.583
Unknown	93.60%			0.42	0.29-0.61	<0.001
**Grade**						
High/Moderate	93.90%	12360.56	<0.001	Reference		
Poor/Undifferentiation	72.30%			2.22	2.12-2.33	<0.001
Unknown	62.90%			2.12	2.02-2.23	<0.001
**TNM**						
I	96.20%	65843.40	<0.001	Reference		
II	87.90%			3.07	2.84-3.32	<0.001
III	76.90%			1.28	1.09-1.51	0.003
IV	17.20%			3.75	3.16-4.46	<0.001
Unknown	67.20%			2.57	2.28-2.90	<0.001
**SEER Stage**						
Localized	94.90%	67229.90	<0.001	Reference		
Regioned	73.20%			4.55	3.91-5.30	<0.001
Distant	15.90%			8.24	7.00-9.73	<0.001
Unstage	52.80%			2.77	2.44-3.14	<0.001
**Marital Status**						
Married	82.10%	1039.78	<0.001	Reference		
Divorced/Separated	77.80%			1.20	1.15-1.26	<0.001
Widowed	70.40%			1.24	1.19-1.30	<0.001
Single	80.10%			1.14	1.09-1.19	<0.001
**Therapy**						
Surgery, radiation or both	86.00%	21427.48	<0.001	Reference		
No surgery, radiation	42.00%			2.09	2.00-2.18	<0.001
Unknown	53.20%			2.25	2.01-2.51	<0.001

### Subgroup analysis for evaluating the effect of marital status on CSS

**Figure 2:**
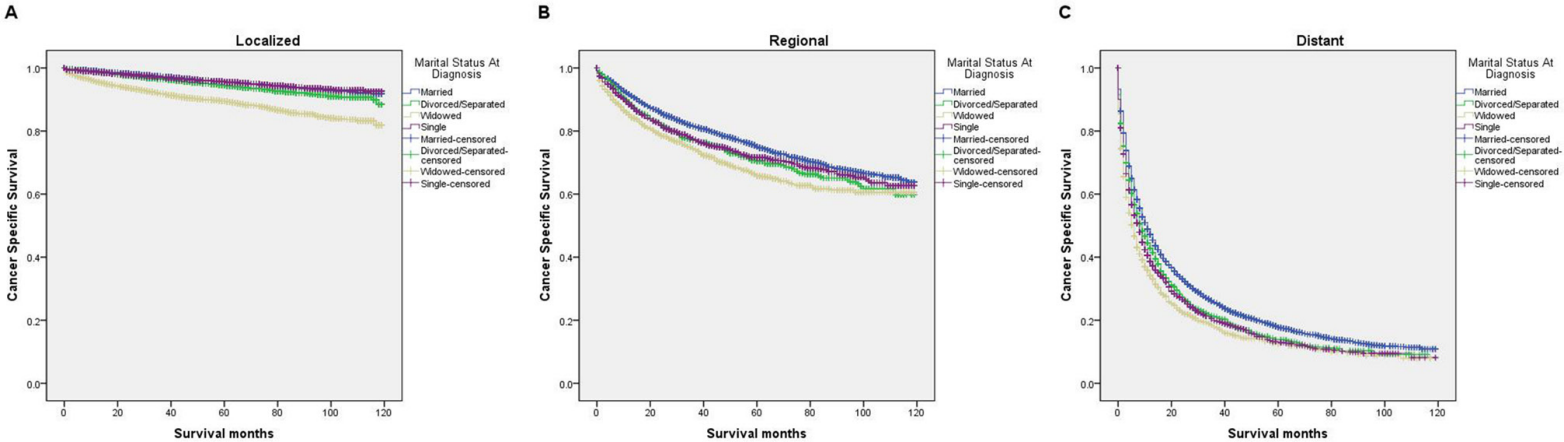
Survival curves in kidney cancer patients on CSS according to marital status in different SEER stages **(A)** Localized: χ2 = 554.76, P < 0.001; **(B)** regional: χ2 = 63.77, P < 0.001; **(C)** distant: χ2 = 198.85, P < 0.001.

**Figure 3:**
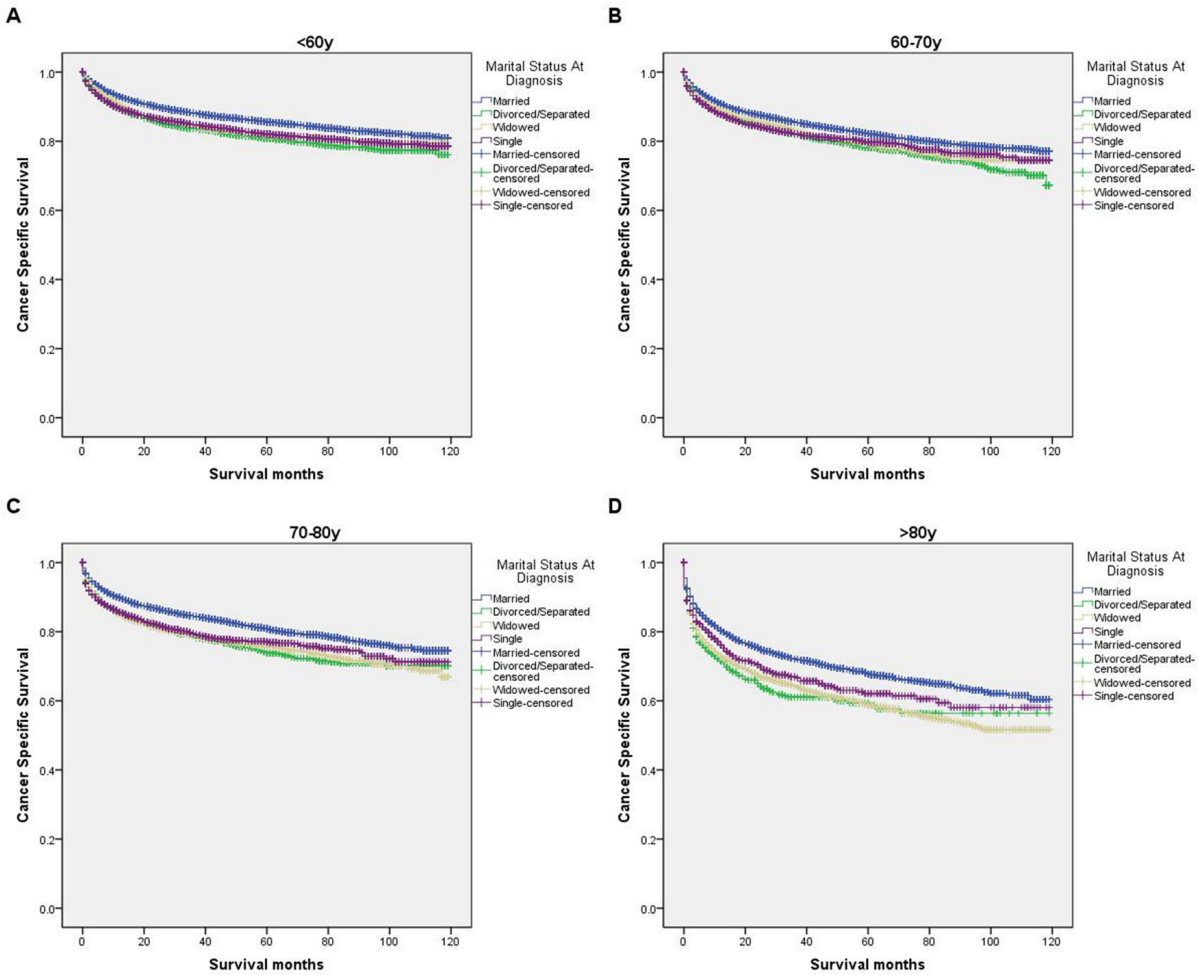
Survival curves in kidney cancer patients on CSS according to marital status in ages **(A)** <60y: χ2 = 116.50, P < 0.001; **(B)** 60-70y: χ2 = 54.83, P < 0.001; **(C)** 70-80y: χ2 = 100.93, P < 0.001 **(D)** >80y: χ2 = 101.93, P < 0.001.

**Figure 4:**
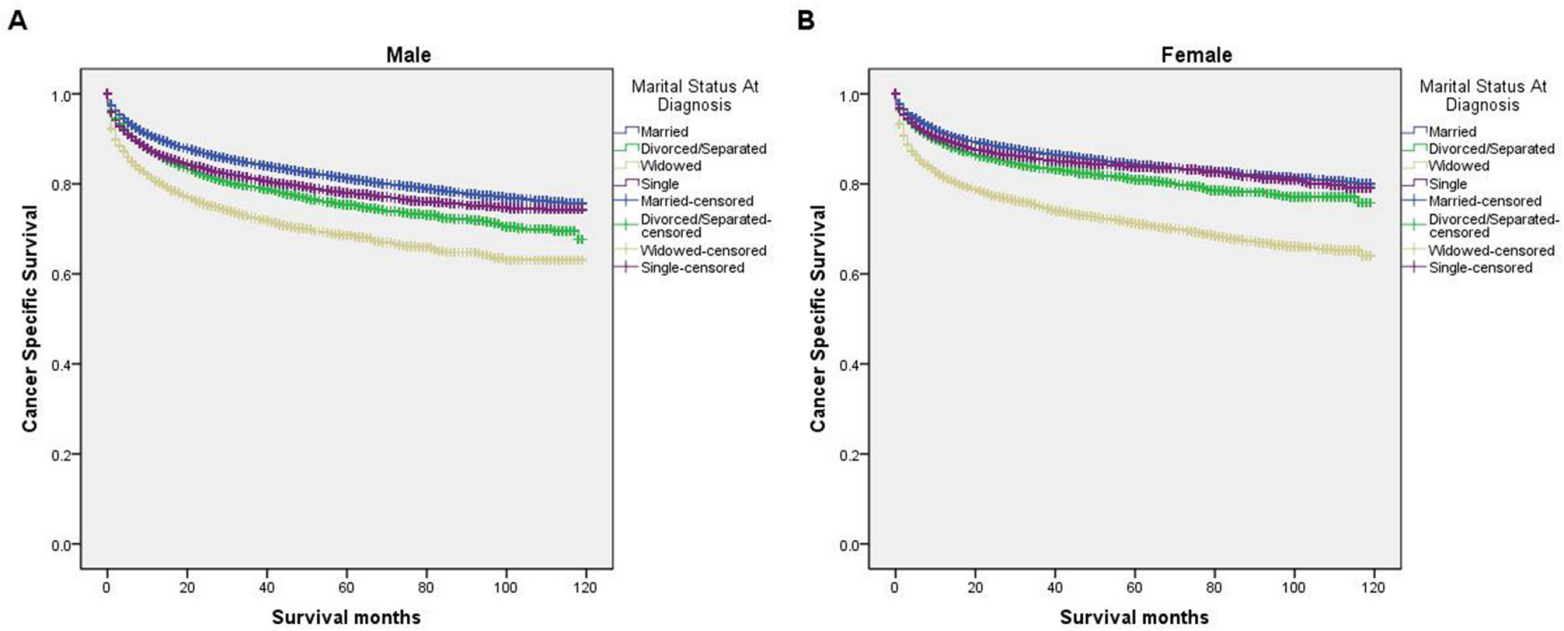
Survival curves in kidney cancer patients on CSS according to marital status in different sexes **(A)** Male: χ2 = 465.71, P < 0.001; **(B)** female: χ2 = 741.71, P < 0.001.

We further explored the effects of marital status on CSS according to SEER stage (Figure 2 and Table 4). Marital status was still an independent prognostic factor at each stage, both in the univariate and multivariate analysis (P<0.001). Moreover, we observed some interesting findings. The widowed patients had poorer CSS in localized, regional and distant stages compared with the other groups, and being widowed was associated with a higher risk of mortality compared with being married in the localized stage (HR=1.51, 95% CI 1.36-1.67, P<0.001) and the distant stage (HR=1.22, 95% CI 1.08-1.37, P<0.001). The survival difference between the single and married groups was not apparent in the localized stage (P=0.679). Furthermore, a previous study showed that prognosis of kidney cancer was associated with increasing age [13]. Therefore, we further analyzed the survival rates and hazard according to age (Figure 3 and Table 5). Married patients always had better survival in each age group, which were consistent with the above results. However, no survival discrepancy was found among divorced/separated, widowed and single patients. We also performed a further stratified analysis by sex. Unmarried patients were shown to have poorer outcomes both in males and females, which was consistent with the above results (Figure 4 and Table 6).

**Table 4:** Univariate and multivariate analysis for evaluating marital status on CSS according to different SEER stage

Variables	5-year CSS	Univariate analysis		Multivariate analysis		
		Log rank Χ^2^	P value	HR	95% CI	P value
**Localized**						
**Marital status**		554.76	<0.001			
Married	95.60%			Reference		
Divorced/Separated	94.50%			1.33	1.18-1.49	<0.001
Widowed	89.50%			1.51	1.36-1.67	<0.001
Single	95.60%			1.15	1.03-1.29	0.013
**Regional**						
**Marital status**		63.77	<0.001			
Married	75.00%			Reference		
Divorced/Separated	70.50%			1.11	0.99-1.25	0.072
Widowed	65.70%			1.06	0.95-1.19	0.311
Single	71.60%			1.11	1.00-1.23	0.050
**Distant**						
**Marital status**		198.85	<0.001			
Married	17.70%			Reference		
Divorced/Separated	13.90%			1.08	0.95-1.22	0.271
Widowed	12.60%			1.22	1.08-1.37	0.001
Single	13.10%			1.20	1.07-1.35	0.002

**Table 5:** Univariate and multivariate analysis for evaluating marital status on CSS according to different age

Variables	5-year CSS	Univariate analysis		Multivariate analysis		
		Log rank Χ^2^	P value	HR	95% CI	P value
**<60y**						
**Marital status**		116.50	<0.001			
Married	85.60%			Reference		
Divorced/Separated	80.80%			1.18	1.09-1.27	<0.001
Widowed	81.80%			1.22	1.03-1.46	0.025
Single	82.10%			1.14	1.07-1.22	<0.001
**60-70y**						
**Marital status**		54.83	<0.001			
Married	82.20%			Reference		
Divorced/Separated	78.30%			1.17	1.08-1.27	<0.001
Widowed	79.40%			1.21	1.08-1.34	0.001
Single	79.80%			1.13	1.03-1.23	0.006
**70-80y**						
**Marital status**		100.93	<0.001			
Married	80.90%			Reference		
Divorced/Separated	73.90%			1.23	1.11-1.37	<0.001
Widowed	75.70%			1.24	1.14-1.35	<0.001
Single	77.00%			1.15	1.03-1.28	0.013
**>80y**						
**Marital status**		101.93	<0.001			
Married	67.70%			Reference		
Divorced/Separated	59.30%			1.25	1.08-1.45	0.003
Widowed	58.80%			1.16	1.08-1.26	<0.001
Single	62.10%			1.07	0.93-1.23	0.328

**Table 6:** Univariate and multivariate analysis for evaluating marital status on CSS according to sex

Variables	5-year CSS	Univariate analysis		Multivariate analysis		
		Log rank Χ^2^	P value	HR	95% CI	P value
**Male**						
**Marital status**		465.71	<0.001			
Married	81.20%			Reference		
Divorced/Separated	75.40%			1.22	1.15-1.29	<0.001
Widowed	68.60%			1.28	1.20-1.38	<0.001
Single	77.90%			1.17	1.11-1.23	<0.001
**Female**						
**Marital status**		741.71	<0.001			
Married	84.30%			Reference		
Divorced/Separated	80.90%			1.16	1.07-1.25	<0.001
Widowed	71.20%			1.14	1.07-1.22	<0.001
Single	83.80%			1.05	0.97-1.14	0.218

## DISCUSSION

In this study, we explored the influence of marriage on OS and CSS in kidney cancer patients based on the SEER program. Our study revealed that married patients had better survival outcomes than unmarried patients, including divorced or separated, widowed, and single patients. In addition, the widowed were at a higher risk of mortality compared with other unmarried groups. Both univariate and multivariate analysis indicated that marital status was an independent prognostic factor for predicting OS and CSS of kidney cancer. Moreover, the survival discrepancy between married and unmarried still existed across different sexes, ages and SEER stages.

The results of our study showed that married patients can enjoy a survival advantage compared with unmarried patients, in agreement with previous studies on other types of cancer [1–4]. However, the mechanisms that drive this correlation are not yet clearly understood. We propose the following underlying mechanisms: Psychologically, depression is frequently observed in cancer patients [14]. Meta-analyses have shown that the depression in cancer patients can increase cancer mortality by 19% to 39% [15, 16]. A recent study showed that depression was significantly associated with a comparatively higher incidence of hospital admissions, emergency department visits, and health system utilization [17, 18]. Married patients have a lower risk of psychological distress, anxiety and depression, as their spouses can help share their emotional burden. Compared with married patients, unmarried patients showed not only greater levels of psychological distress but also lower levels of the fighting spirit and higher levels of helplessness [19]. Cancer survivors in the United States reported medication use for anxiety and depression at rates nearly two times those reported by the general public, and unmarried are more likely to use medication [20]. In addition, psychological stress may cause poorer adherence to treatment [21].

Physiologically, marriage may have direct influences on cardiovascular, endocrine, immune, neurosensory, and other physiological functions [22]. Endocrine hormones, such as cortisol and catecholamine, can promote tumor growth and metastasis [23]. Married individuals had lower cortisol levels than their never married and previously married counterparts. Cortisol could be regarded as one candidate mechanism accounting for the association of marital status and health [24]. Additionally, the hypothalamus-pituitary-adrenal axis may also have an effect on the immune system, leading to poorer survival [25, 26].

Another widely accepted explanation of why married patients have better prognosis of cancer and other diseases is that married patients have better social-economic support. It has been reported that married persons are more likely to have better access to medical care and more financial support than unmarried persons [8]. In addition, married couples may have wider health insurance coverage [27]. Thus, spouses might contribute to earlier disease detection [8]. These factors could partly explain why married patients corresponded to a higher percentage of earlier stage cancer in our study. Married patients also have better adherence with prescribed treatments. We found that unmarried, especially widowed, had a lower percentage of surgery and radiotherapy, which could be attributed to the survival difference between the married and unmarried patients. In addition, lifestyles, such as diet habit and behaviors, may also be influenced by marriage [28]. For example, it is well accepted that smoking increases the risk of kidney cancer. Studies show that living without a spouse increases daily smoking rates [29]. Moreover, marriage is associated with an increased probability of cessation for men [30].

Nevertheless, some limitations should be noted. First, the SEER database only provides marital status at the time of diagnosis, which may change after diagnosis. In addition, the quality of the marriage could not be evaluated in the follow-up. SEER also does not record cohabitation status, patients cohabitating with their partners may be categorized as unmarried in the SEER database. Second, we had no direct information on socioeconomic factors, such as educational information, income status, insurance status, and lifestyle (such as smoking and alcohol use), which are not recorded in the SEER database. Third, the SEER database is unable to provide other important survival factors, such as chemotherapy, details of surgery, and other types of therapy. Lastly, as a retrospective study, we could not avoid various forms of bias.

Despite these potential limitations, our study revealed that married kidney cancer patients had survival advantages, while unmarried patients were at higher risk of overall and cancer-specific mortality. We supposed that psychological, physiological and social-economic factors may contribute more to survival outcomes among married patients. Physicians should realize this significant survival difference according to marital status. More social support services and medical interventions should be provided for unmarried patients. Furthermore, future studies should focus on the mechanisms among marital functioning, physiology, and health and on genetic and other variable explanations for marriage-related health outcomes. Additional studies of objective social behavior after changes in marital status are also warranted.

## MATERIALS AND METHODS

### Data source and patient selection

This study used data from the SEER database released in March 2017, which covered approximately 28% of the United States population. By using SEER-stat software (version 8.3.4), we searched the database for patients diagnosed between 2004 and 2013 with kidney cancer. Patients aged 18 or older at the time of diagnosis with kidney cancer (International Classification of Diseases for Oncology, Third Edition [ICD-O-3], code C64.9) were included for analysis. Patients were excluded if they had unknown marital status, unknown cause of death or unknown survival time. A total of 112860 patients were included in the cohort. This study was based on public data from the SEER database; we obtained permission to access research data files with the reference number 14920-Nov2015. Because this study did not include the use of human subjects or personal identifying information, this study did not require informed consent.

### Study variables

Variables extracted from the SEER database included marital status, sex, age at diagnosis, race, histological grade, TNM stage, SEER stage, and selection of therapy (surgery/radiotherapy). Marital status is coded as married, divorced or separated, widowed, and single. Age at diagnosis was divided into four groups: <60 years, 60-70 years, 70-80 years, and ≥80 years. Race was classified as white, black, American Indian/Alaska Native, Asian/Pacific Islander, or unknown. Histological grade was classified as well/moderately differentiated, poorly differentiated/undifferentiated, or unknown. SEER stage was categorized as localized, regional, distant, or unknown. Selection of therapy was divided into three groups: surgery or radiation or both, no surgery or radiation, and unknown.

### Outcomes

The primary outcomes were OS and CSS. OS was calculated from the date of diagnosis to the date of death from any cause. Patients who were alive on the date of last contact or at the follow-up cut-off date were censored. CSS was defined as the time from diagnosis to the date of death due to kidney cancer. Death caused by kidney cancer was considered an event. Patients who died from other causes or who were still alive at the time of the last follow-up were treated as censored. The follow-up cut-off date was December 31, 2013, according to the SEER database.

### Statistical analysis

The association between marital status and clinicopathological features was assessed by the chi-square (χ2) test. Survival curves were generated by the Kaplan-Meier method; differences between the curves were analyzed by using the Log-rank test. Multivariable Cox proportional hazards regression models were used to assess potential risk factors for survival outcomes. All statistical analyses were performed using SPSS for Windows, version 19 (SPSS Inc., Chicago, IL, USA). All P values were 2-sided, and P<0.05 was considered statistical significance.
